# Unusual Case of Urethrorectal Fistula in Adolescence in a Patient with a History of Congenital Anorectal Malformation

**DOI:** 10.1089/cren.2015.0042

**Published:** 2016-02-01

**Authors:** Constantinos Nastos, Ira Sotirova, Athanasios Papatsoris, Andreas Skolarikos, Ioannis Papaconstantinou, Athanasios Dellis

**Affiliations:** ^1^ Second Department of Surgery, Medical School, National and Kapodistrian University of Athens, Aretaieion University Hospital, Athens, Greece.; ^2^ Second Department of Urology, Medical School, National and Kapodistrian University of Athens, Sismanogleion Hospital, Sismanogleiou, Greece.

## Abstract

***Background:*** Urethrorectal fistula is a rare and debilitating condition. Spontaneous closure is rarely effective, and appropriate management regarding timing of repair and surgical approach remains controversial.

***Case Presentation:*** We present a case of an 18-year-old male found to have a urethrorectal fistula after diagnostic work up for unejaculation. The patient gradually developed recurrent urinary tract infections and urine and semen leak from his rectum. He had a medical history of an anorectal reconstruction in the second postnatal day due to an anorectal malformation. Imaging with a rectal endoscopic ultrasound scan revealed a suprasphincteric urethrorectal fistula that was further confirmed with semirigid urethrocystoscopy and placement of a nitinol guidewire through the urethral fistula orifice. Its anal orifice was 3 cm above the anal verge at the 12th hour of the rectum. The fistula orifice on the rectum was identified with a transanal approach and the fistula was managed with the performance of an advancement mucosal flap and bladder catheterization. The patient developed a recurrence with this approach and finally underwent fistula ligation and reconstruction using a scrotal flap. The patient has not had a recurrence of the fistula during his follow-up.

***Conclusion:*** This is an unusual case of iatrogenic urethrorectal fistula as it presented in adolescence many years from the initial operation of anorectal reconstruction and with unusual symptoms.

## Introduction

Urethrorectal fistula is a rare yet potentially debilitating condition, which can affect both males and females in all age groups. Urethrorectal fistulas may be suspected when classical symptoms, such as fecaluria, pneumaturia, abnormal urethral discharge, or leakage of urine from the rectum during micturition, are present. In our case, the first manifestation of the fistula was unejaculation during masturbation, since there was no sexual life yet, and it occurred during adolescence. We report a case of a late presenting urethrorectal fistula in an 18-year-old male who underwent anal reconstruction early after birth and developed the first symptoms and signs of the fistula in adolescence.

## Case Presentation

### Clinical history

An 18-year-old male was evaluated in the outpatient clinic of our hospital complaining of unejaculation during masturbation for the last several years. From his medical history, it was noted that he was born with a low anorectal malformation, which was surgically treated on the second postnatal day with anorectal reconstruction. Pregnancy was full term with normal delivery and uncomplicated otherwise. The recovery period was uneventful. The patient was subjected to multiple anal sphincter dilatations using Hegar dilators (size 12) for the first 6 months after the operation. For the next 17 years, he suffered from severe constipation, which was treated conservatively, and occasional episodes of soiling. He never developed any acute or chronic urinary tract infections (UTI) until the age of 17 and remained sexually inactive. At the age of 17, he started complaining of recurrent episodes of UTIs from *E. coli* that were treated with oral ampicillin. These episodes were followed by urine and semen leak from the rectum after masturbation and patient thought these symptoms to be related to incontinence. In addition, after masturbation the patient failed to ejaculate.

### Diagnosis

**FIG. 1.**
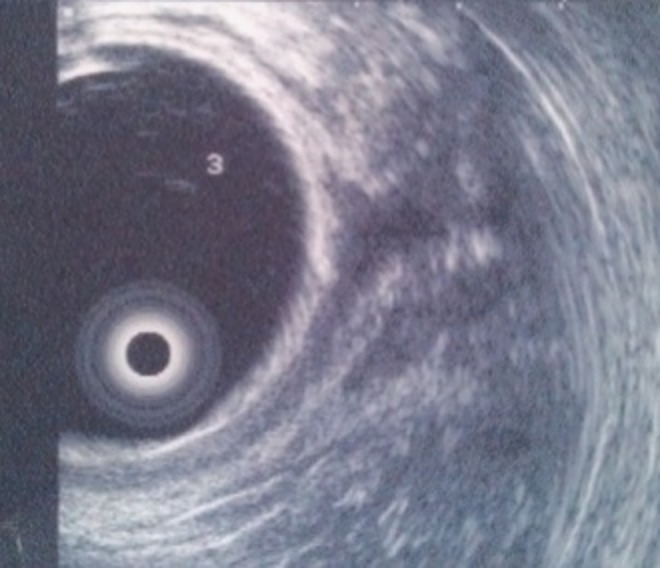
Endoscopic ultrasound image of the fistula. The fistula tract is depicted in the 12th hour of the rectum, suggesting a suprasphincteric fistula 3 cm above the anal verge.

**FIG. 2.**
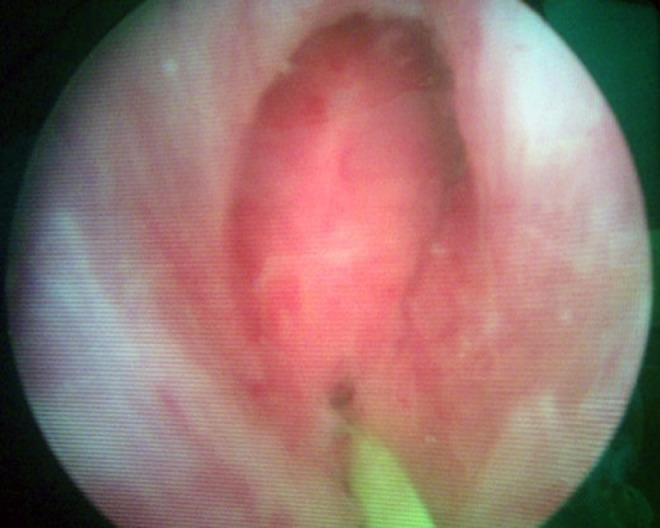
Fistula orifice right distal to the verumontanum and proximal to the striated sphincter.

The patient was initially elsewhere investigated with a flexible cystourethroscopy, a retrograde cystourethrography, MRI of the pelvis, and rectosigmoidoscopy without any abnormal findings. A digital rectal examination did not reveal any perineal fistula or any other abnormal findings. However, because of his persisting symptoms the patient underwent additional imaging with anorectal endoscopic ultrasound scan, which revealed the presence of a suprasphincteric fistula 3 cm above the anal verge and at the 12th hour of the rectum, leading to the diagnosis of a rectourethral fistula (Fig. 1). Semirigid cystourethroscopy revealed a small diverticulum on the left ureteral orifice and a fistula orifice at the 6th hour of the urethra right distal to the verumontanum and proximal to the striated sphincter (Fig. 2). The opening was catheterized with a nitinol zip wire (Boston Scientific, Natick, MA) and a connection was identified with the rectum as the wire exited through the rectum and anus.

### Intervention

Following the diagnosis of urethrorectal fistula, the patient was subjected to transanal rectal advancement flap repair under general anesthesia. The transanal Latzko technique was chosen, using the nitinol zip wire for identification of the fistula orifice on the rectum, because it is effective, simple to perform, and has minimal morbidity. The Latzko technique does not involve the section of the anal sphincter in contrast with the transrectal trans-sphincteric approach (York-Mason technique).^1^ The patient was placed in the lithotomy position. Cystourethroscopy was performed and the urethral fistula orifice was reidentified and catheterized until the entire course of the tract was followed and the guidewire protruded through the anus. The rectal opening of the fistula was exposed and identified under direct vision at the 12 O'clock position. After injection of adrenaline solution to the submucosa surrounding the fistula, a flap of mucosa, submucosa, and part of the muscular layer was created and covered the rectal side of the fistulous tract after excising the fistula orifice to the rectum. The patient had an uneventful recovery and was discharged on the first postoperative day with a 16F all-silicone indwelling urinary catheter.

### Follow-up and reintervention

Follow up was arranged in the 20th postoperative day with voiding cystourethrography that revealed no leakage from the urinary bladder or urethra and finally the catheter was removed. Despite the uneventful postoperative period and the normal imaging, the patient presented recurrence of the fistula 1 month after removal of the urinary catheter with the same clinical manifestations. He was further managed with scrotal flap reconstruction. The patient was placed again in the lithotomy position. Cystourethroscopy was performed and the urethral fistula orifice was reidentified and catheterized with a nitinol guidewire that protruded through the anus. A midline “inverted Y” perineal incision was performed. The fistula was well prepared circumferentially in its whole length and excised with urethral and rectal orifices included. Urethral and rectal defects were closed separately with running absorbable 5/0 sutures. A ventral scrotal skin flap was prepared and de-epithelialized with scissors so as to avoid damage of its vasculature and finally interposed between the urethra and rectum. The perineal skin was reapproximated with nonabsorbable 3/0 sutures. Again the patient had an uneventful recovery and was discharged on the second postoperative day with a 16F all-silicone indwelling urinary catheter and a suprapubic catheter. The postoperative follow-up consisted of urine cultures at the first and third month after catheter removal of the final procedure as well as voiding cystourethrogram at the third postoperative month. He does not have a recurrence 2 years after the final procedure.

## Discussion

Urethrorectal fistula is a rare and unpleasant complication that is managed with reconstructive procedures, because spontaneous closure is rare.^2^ In addition, appropriate management regarding timing of repair and surgical approach remains controversial.^3^ The etiology varies, but can be classified as congenital, iatrogenic, traumatic, neoplastic, and inflammatory. As much as 60% of cases are thought to be iatrogenic.^4^ Congenital fistulas are rare and are most commonly associated with anorectal malformation disorders. In these patients, fistulas either coexist with the malformation of the anus and rectum or can be the results of the surgical correction of the malformation. There are reports of co-existing fistulas being missed during the work up of the anorectal malformation disorder and symptoms occurring many years after, during the adult life. However, these cases are extremely rare.^1^

In our case, there is no way of knowing if the fistula was congenital or the result of either the initial operation for the correction of the anorectal malformation or the subsequent multiple dilatations. It is rather interesting and surprising that the clinical manifestations of the fistula became apparent after more than 15 years from the initial operation. In retrospect, there were no convincing symptoms that could be linked to the existence of the fistula during childhood or adolescence. In contrast, it is unlikely that the fistula developed during this period and most probably it existed from the perinatal period. This assumption is in agreement with other case reports, where there were delayed clinical manifestations of the congenital fistulas during adult life.

There is a wide spectrum of symptoms that can be associated with urethrorectal fistulas. The usual presentation is frequent and recurrent UTIs, combined with fecal discharge during urination and urinary discharge during defecation. In our case, the cause that led to the investigation of the patient was symptoms similar to retrograde ejaculation after masturbation, which is a rather rare manifestation of urethrorectal fistula. The explanation of this manifestation is the location of the urethral orifice of the fistula. Its location right distal to the verumontanum and proximal to the striated sphincter was probably the reason for this manifestation.

The choice of surgical technique for the management of the urethrorectal fistula is a challenge for the surgeon. The dilemmas in choosing the correct surgical technique are due to the high incidence of fistula recurrence.^1^ There are no prospective studies due to the rarity of the disease, and in the literature there are only case reports and small case series.^1^ Various techniques have been adopted for the treatment of urethrorectal fistula, including perineal, abdominal, and mixed approaches. The transabdominal approach is more difficult and complicated with high morbidity, prolonged postoperative recovery, and limited success. The perineal approach involves rectal advancement flap repair. Excellent results have been demonstrated with this technique, particularly in combination with interposition of flaps from other tissues, such as levator ani muscle, gracilis muscle, buccal muscle, and others.^1^

A recent systematic review, including data from all patients reported by various case series and case reports, showed that the failure rate of the transanal approach (Latsko procedure) was 41% in a total of 22 patients. Closure of urethrorectal fistula with conservative treatment was rare. The laterosacral or posterior approach (Kraske procedure) can also be useful, but is more practical for surgical treatment of presacral tumors. The trans-sphincteric approach (York-Mason) is a posterior approach, in which all layers of the anorectal sphincter are divided for direct access to the fistula, located at the anterior rectal wall. Failure was reported in 12% of the patients treated with this approach. Although this method has good results for the treatment of the fistula, it involves section of the anal sphincter.^1^

## Conclusion

Urethrorectal fistula is a rare yet potentially debilitating condition representing a challenge for the surgeon and the patient because spontaneous closure is rare and there is no consensus in the literature regarding the treatment of these fistulas. The surgeon should choose the best approach for the individual case. The goal of the present case report was to demonstrate unusual and late presentation of urethrorectal fistula after surgical management of a congenital anorectal malformation and the precise endourologic arm of diagnostic procedures since initial work up failed to demonstrate the fistula existence.

## Abbreviations Used

MRI: magnetic resonance imaging
UTI: urinary tract infections
